# Predicting Low-Modulus Biocompatible Titanium Alloys Using Machine Learning

**DOI:** 10.3390/ma16196355

**Published:** 2023-09-22

**Authors:** Gordana Marković, Vaso Manojlović, Jovana Ružić, Miroslav Sokić

**Affiliations:** 1 Institute for Technology of Nuclear and Other Mineral Raw Materials, 11000 Belgrade, Serbia; g.markovic@itnms.ac.rs (G.M.); m.sokic@itnms.ac.rs (M.S.); 2 Faculty of Technology and Metallurgy, University of Belgrade, 11000 Belgrade, Serbia; 3 Department of Materials, “Vinča” Institute of Nuclear Sciences—National Institute of the Republic of Serbia, University of Belgrade, 11000 Belgrade, Serbia; jruzic@vin.bg.ac.rs

**Keywords:** titanium alloys, machine learning, Extra Tree Regression, Monte Carlo method, Young’s modulus

## Abstract

Titanium alloys have been present for decades as the main components for the production of various orthopedic and dental elements. However, modern times require titanium alloys with a low Young’s modulus, and without the presence of cytotoxic alloying elements. Machine learning was used with aim to analyze biocompatible titanium alloys and predict the composition of Ti alloys with a low Young’s modulus. A database was created using experimental data for alloy composition, Young’s modulus, and mechanical and thermal properties of biocompatible titanium alloys. The Extra Tree Regression model was built to predict the Young’s modulus of titanium alloys. By processing data of 246 alloys, the specific heat was discovered to be the most influential parameter that contributes to the lowering of the Young’s modulus of titanium alloys. Further, the Monte Carlo method was used to predict the composition of future alloys with the desired properties. Simulation results of ten million samples, with predefined conditions for obtaining titanium alloys with a Young’s modulus lower than 70 GPa, show that it is possible to obtain several multicomponent alloys, consisting of five main elements: titanium, zirconium, tin, manganese and niobium.

## 1. Introduction

Titanium and its alloys have played a significant role in the medical industry since the 1960s [1]. They have been used in the production of various medical devices, including artificial heart valves, vascular stents, and orthopedic implants such as artificial shoulders, hips, and knees [2,3]. In the field of dental implants, titanium and its alloys are considered strong and efficient components [4]. High strength, excellent biocompatibility and corrosion resistance, low density, a low Young’s modulus (*E*), and good osseointegration are the most desirable properties of the titanium and its biocompatible alloys. There are many definitions dealing with the concept of biocompatibility, but the most general one would be that it is the ability of the implant to perform the desired function with an appropriate host response. There are five criteria established by the international standards organization, and they say that the implant must be non-toxic, non-thrombogenic, non-carcinogenic, non-antigenic and non-mutagenic for the body [5,6]. In the case of titanium alloys, the toxicity is related to the release of the element into the body fluids, due to corrosion-induced deterioration of the material. Due to their superior biocompatibility, Ti alloys are ideal for use in body fluids that tend to change pH values [7]. Furthermore, they are exposed to varying amounts of stress on a daily basis, including the extreme levels of stress experienced by normal hip joints during physical activity [8]. As a result, corrosion-resistant and high-strength titanium alloys have proven to be an excellent option [9]. Although stainless steels and cobalt alloys can also be used for similar applications, titanium alloys are favored because of their significantly lower Young’s modulus and higher specific strength [10]. Therefore, they have become the primary choice in orthopedic and dental implants more often than any other group of metals [1].

Titanium possesses two allotropic forms which depends on temperature, the alpha phase (*α*, hexagonal close-packed crystal structure) at lower temperatures, and beta phase (*β*, body-centered cubic crystal structure) at higher temperatures, where the transformation temperature of the *α* into the *β* is at 1155 K [11]. Alloying elements added to pure titanium with possibility to promote stability of the *α*-phase are called *α*-stabilizers, while those that promote the stability of the *β*-phase, are classified as *β*-stabilizers (Table 1). Alloying elements with negligible effect on the stability of the *α*- and *β*-phases are known as neutral (Table 1). Both the type of alloying element added and the amount in which it is added have a significant effect on the titanium microstructure. In general, titanium alloys are categorized into six groups according to the content of the alloying elements and their tendency to form *β*-phase [11]: *α*-alloys, near *α*-alloys (1–2 wt.% *β*-stabilizers), *α* + *β* alloys (4–6 wt.% *β*-stabilizers), metastable *β*-alloys (10–15 wt.% *β*-stabilizers), *β*-alloys (30 wt.% *β*-stabilizers), and titanium aluminide—intermetallic alloy (20–45 wt.% *β*-stabilizers).

Pure titanium (*α*-alloys) and Ti-6Al-4V (*α* + *β* alloy) are the most widely used alloys in biomedical engineering [12]. Recent studies reviled that *β*-alloys could be used as biomaterials owing to their excellent biocompatibility, high strength, and low *E* as well [13]. The reason for questioning the safety of implants made of Ti-6Al-4V alloy is the possibility of vanadium and aluminum ions being released as a result of corrosion and their negative impact on the organism in which it is implanted. Some previous studies have attributed these ions to a variety of health problems and diseases, including brain damage, breast cancer, anemia, digestive disorders, neurotoxicity, contact dermatitis, male fertility, and Alzheimer’s disease [13,14]. It is important to note that no material is completely bioinert, and careful selection of alloy components is essential [11]. The classification of alloying elements in terms of their effects on the organism (non-cytotoxic, neutral and cytotoxic elements) are given in Table 2 [15]. In addition elements that are considered cytotoxic, it is important to note that some non-cytotoxic elements have negative impact on the organism due to their higher concentration or being placed in different environments. All mention above indicates that careful design of a novel materials which undergo comprehensive and long-term testing is necessary for manufacturing a human-safe implant. Another challenge lies in the fact that Ti-6Al-4V has a higher *E* (110 GPa), compared to bone (<35 GPa), which results in uneven stress transfer between the implant and the bone. This can lead to bone atrophy and implant failure [16,17].

Moreover, a factor to be taken into account is the constant and accelerated growth of the population, as well as the increase in life expectancy. Accordingly, it is estimated that in the next few years the number of performed procedures for the implementation of Ti-6Al-4V alloy implants will be several million, and the main users will continue to be the elderly population [13]. It is also important to pay attention to the price of raw materials [18]. This aspect is usually approached from two directions, using more expensive raw materials in small quantities or using cheaper raw materials if the alloying element occurs in larger mass fractions [19]. Recently, emphasis has been placed on obtaining information about the properties of multicomponent alloys whose compositions correspond to the central parts of the ternary and quaternary systems [20]. According to everything mentioned so far, it is clear that there are new, more complex requirements for the design of biocompatible titanium alloys, which are characterized by a low Young’s modulus and a low price.

In recent years, machine learning is widely used in material science for development and design of novel materials with specific properties. Among numerous computational approaches, the most commonly used empirical calculation methods for designing low Young’s modulus titanium alloys, are: the molybdenum equivalence (*Mo_eq_*) method, the method of the ratio of electrons to atoms (*e/a* ratio) versus the difference in diameters, the d-electron theory where design method is based on d electrons, and the high-throughput method [15]. Even though these models provided broad knowledge and understanding of various phenomena in material science and other fields, they are often inefficient due to complex computational requirements and costly equipment. Machine learning proved to be a new approach that could be applied in material science for efficient prediction of material properties, and their chemical compositions. Hence, in recent years the correlation between chemical composition and low-modulus of elasticity of biocompatible titanium alloys successfully established by machine learning methods was objective of several studies [15,21].

In this study, machine learning techniques were employed to determine underlying relationships that govern the mechanical properties of biocompatible Ti alloys, specifically their elastic behavior. The used computational approach consists of an established database of biocompatible Ti alloys, conducted an exploratory data analysis, created new feature classifications based on solid scientific principles, and the regression model. The Extra Tree Regression method was identified as the most effective machine learning model for predicting the *E* with the deviation between experimental and predicted data remaining within the acceptable range as specified in the literature. The novelty of this study is that the specific heat was recognized as the parameter exerting the most substantial impact on the *E*. Furthermore, distinct compositional ranges have been established for a four-component alloy that maintains a low *E*, where all the constituents act as *β* stabilizers. Importantly, these elements rank among the most cost-effective alloying options, making them suitable for wider applications. Obtained results were facilitated by the use of the experimental design methodology to construct multidimensional, four-component diagrams.

## 2. Materials and Methods

**Figure 1:**
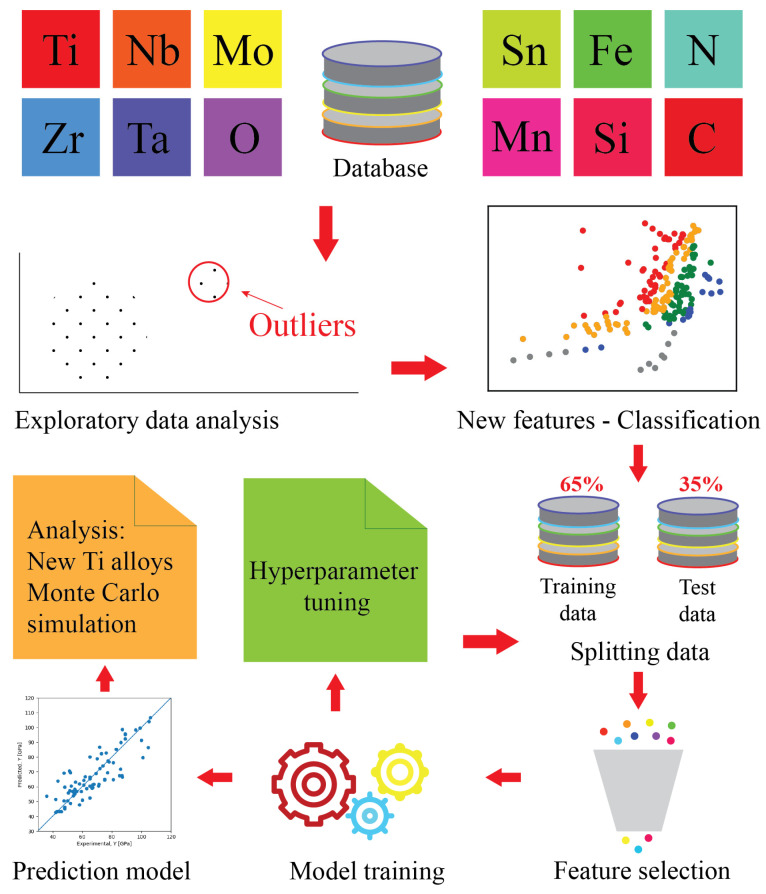
Computational approach employed to identify influential parameters on the Young’s modulus of biocompatible Ti alloys and to predict their composition.

Computational approach employed in this study is illustrated in Figure 1. It contains several steps as follows: establishing a database of biocompatible Ti alloys, conducting an exploratory data analysis, building new feature classifications according to solid scientific principles, and identifying appropriate regression model for predicting the composition of biocompatible Ti alloys with a low Young’s modulus.

### 2.1. The Database

A database consisting of 246 biocompatible titanium alloys was used, with experimental data for *E*, alloy composition, mechanical and thermal treatment (literature based). The script for calculated parameters is given in the Zenoodo platform [22]. Detailed explanation of parameters and procedures is given in the publication related to this database [23].

### 2.2. Data Preprocessing

The prediction model is a powerful tool used to estimate Young’s modulus from available data on alloy compositions [24]. The development process involves precise data preparation, which includes careful selection of relevant columns and identification of outliers. Through this data preparation process, the prediction model is equipped with higher quality data, allowing more accurate estimates of *E* for different alloy compositions. In this context, irrelevant columns which do not contribute to the prediction task are removed, thus simplifying the dataset and reducing complexity. Outliers, which can potentially skew model performance, are detected using techniques such as scatter plots and histograms. Various outlier detection methods, including linear regression, Z-score method, interquartile range (IQR) method, local deviation factor method combined outliers, are used to identify and process deviations from standard values in a dataset [25,26].

Incorporating scientific principles, new features were introduced to enhance the predictive efficacy of our model. Two illustrative diagrams were constructed to elucidate the relationships between various factors. The first, termed the “*Bo-Md*” diagram, displays the relationship between the d-orbital energy level (*Md*) and the Bond order (*Bo*) between titanium and the alloying elements. Utilizing the coordinates from this diagram, the data were classified into distinct categories according to the identified phases present, corresponding deformation mechanisms, and respective Young’s modulus values within each alloy set (class 1: slip *β*, class 2: twin (*β* + *ω*), class 3: marteniste (*α*′ + *α*″), class 4: *α* + *β*, class 5: *α*).

The second diagram delineates the electron-to-atom (*e/a*) ratio, offering insights into the differences in atomic radii and thereby indicating the deformation mechanisms at play. A similar categorization process was applied using the coordinates obtained from this diagram, leading to the formation of two distinct data groups (split and twin).

The next step involves feature selection, where the aim was to identify the most relevant and influential features that are significantly related to the *E*. By selecting these relevant features, we can reduce the complexity of the model and focus on the characteristics that have a strong association with the target variable. Through an iterative process of elimination, we systematically assessed the impact of each feature on the model’s performance. Any feature that did not enhance or adversely affected the model’s performance, as indicated by the observed metrics, was removed from the training model. Consequently, only those features that positively influenced the model’s performance were retained. The ‘feature importance’ technique was also used to select most important features, as described in the literature [27].

Following data preprocessing and feature selection, the dataset was divided into two subsets for model training and testing. A total of 65% of the data was assigned for training purposes, while the remaining 35% was reserved for testing the model’s performance. In machine learning practice, allocating 35% of the data for testing is considered a relatively large portion, which, in our case, given the small size of the dataset, ensures a robust evaluation of our model’s predictive performance. In order to scale the features to a standard range and prevent any single feature from dominating the learning process of the model, normalization was used, namely MinMaxScaler [28]. By applying data partitioning and normalization, we can create a reliable and unbiased evaluation framework for a prediction model, allowing us to accurately assess its performance and make meaningful predictions on new, unseen data. A random state parameter with a value of 42 was also used in order to enable reproducibility and consistency of the results. Interventionary studies involving animals or humans, and other studies that require ethical approval, must list the authority that provided approval and the corresponding ethical approval code.

### 2.3. The Prediction Model

The most appropriate prediction model was determined using the “lazy prediction” approach. This methodology assesses multiple models and select the one that best suits specific task, thus ensuring the most reliable and accurate predictions for particular study [29]. The performance of models trained on the training data was determined for 42 prediction models, with the Extra Tree Regressor performing best [30]. The performance of the Extra Tree Regressor is validated for both the training set and the test set, as well as a visual representation of the predicted relationship.

#### Metrics

Metrics are quantitative measures used to evaluate the quality of a model. The following metrics were used in this paper: *MAE* (mean absolute error), *MSE* (mean square error), *MAX* (maximum absolute error), *MAPE* (mean absolute percentage error) and *RMSE* (root mean square error). Additionally, adjusted *R*^2^ and *R*^2^ were used. The difference being that adjusted *R*^2^, based on the number of predictors and sample size, explains how well the independent variables explain the variability of the dependent variable. *R*^2^ does not take into account the number of predictors [31,32].

### 2.4. Visualization of Results

For the analysis of individual and combined effects of alloying elements on the *E*, double diagrams were made. The range of values of the mass fractions of the elements was determined by calculating the maximum and minimum values of the corresponding column from the training set, and by combining the values a data grid was obtained. With the help of predictions, each point on the grid is assigned a certain value for the *E*, based on which different regions of values are formed, which are connected to the scales on the right. *Bo-Md* diagrams were formed in the same way, as well as the ratio *e/a–*Δ*r* [33]. It provided a visual overview of which are the most optimal ratios of independent variables that can give the desired values for the *E*.

### 2.5. Experimental Design for Predicting the New Alloys

Experimental design is a method that involves planning and conducting experiments in a non-traditional way that requires a quick and efficient approach. The factors that will be analyzed are selected—in our case, they are the mass fractions of alloying elements and the ranges are defined for each of them (minimum is 4, and maximum 40 wt.%, that appeared in the database for each of the alloying element). Approximately 16,000 different alloys were generated using this approach.

To forecast the composition and characteristics of multicomponent alloys, a Monte Carlo simulation was employed. This technique, known for generating random alloy compositions, facilitated the execution of ten million different alloys. This comprehensive computational approach enabled a broad exploration of potential alloy compositions, thereby supporting the robust prediction of a low Young’s modulus using developed model [34].

## 3. Results and Discussion

### 3.1. Basic Statistics, Data Correlation

For 49 variables, as defined in the database, the values of the following parameters were determined: minimum, mean, maximum, standard deviation, and median. The second column represents the number of parameters that were available for each of the variables (Table A1).

In the dataset, one notable observation is the markedly low minimum value for the *E* for several alloys. This value falls significantly below the median, indicating an extreme deviation that demands careful consideration during model development, as it has the potential to influence the precision of our model performance.

It is important to note that deviations in the Young’s modulus primarily arise due to variations in thermal and mechanical treatment across dataset [35,36]. However, owing to the incomplete data for these factors in the current dataset, we were unable to incorporate this information into our machine learning model at this stage. Future efforts to collect and include data pertaining to thermal and mechanical treatments would be crucial in enhancing the predictive performance of our model, thus providing a more accurate representation of the modulus of elasticity for various alloys.

Additionally, high standard deviation values in the case may indicate that some points are significantly further from the mean. Standard deviation data alone are not sufficient for a general conclusion, but it can be a good predictor when it comes to points that may influence further statistical analyses.

**Figure A1:**
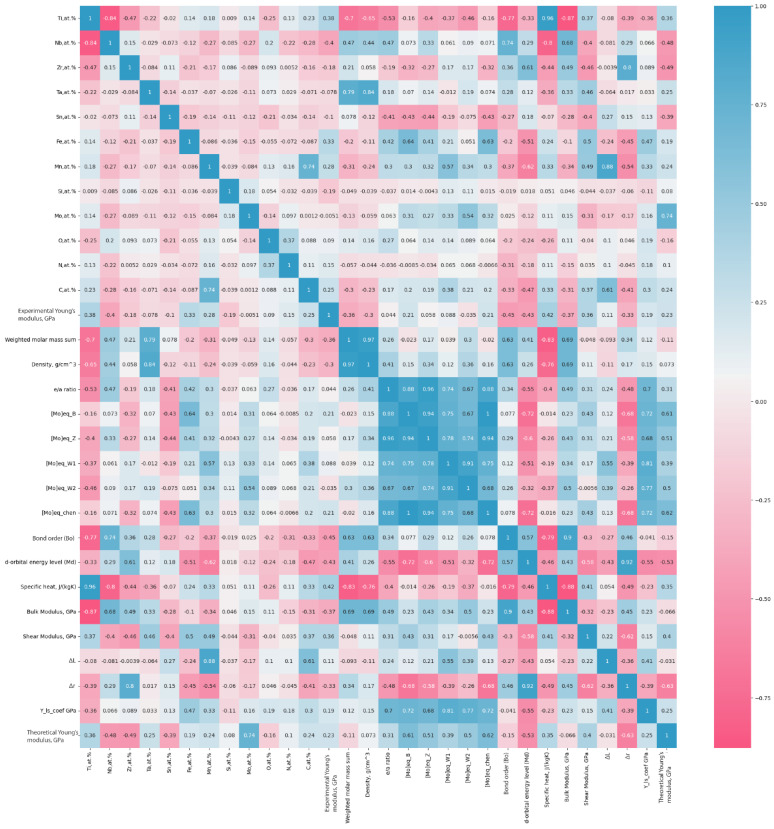
Correlation matrix of independent variables.

A correlation matrix was generated to examine the relationships between pairs of variables in the dataset (Figure A1). Correlation coefficients, ranging from −1 to 1, are visually represented using a spectrum of colors from intense pink to white to dark blue. A coefficient of −1 indicates a strong negative correlation, while a coefficient of 1 indicates a strong positive correlation. Values close to 0 indicate no correlation. This analysis is particularly important in identifying multicollinearity, as high correlations between variables can lead to unreliable predictions and make it difficult to determine the individual effects of factors on outcomes.

High collinearity was observed for the mass fractions of the following pairs: C-Mn i is 0.74 and O-N is 0.42. Carbon in titanium alloys is mainly found as an impurity, while manganese is an alloying element. Their high correlation coefficient can be explained by a similar influence on the grain refinement, whereby a fine-grained and therefore stronger alloy is obtained [36]. Oxygen and nitrogen are mainly impurities in titanium alloys that are introduced with the raw material itself or during processing; however, they can have a very similar effect, which is known through the collinearity coefficient. Both elements in small amounts can increase the strength of the alloy, precipitate oxides formed by oxygen and nitrides formed by nitrogen. Additionally, when it comes to corrosion protection, the oxygen that forms the oxide layer acts as a barrier, while nitrides are especially important in aggressive environments [37].

### 3.2. Making New Features Using Classifications

#### 3.2.1. Classification Based on: D-Orbital Energy Level (Md) vs. Bond Order (Bo)

The Bond order is a measure of the strength of the covalent bond, while the energy level of the d-orbitals of transition elements is influenced by their metallic radius and electronegativity. The average *Bo* and *Md* values of alloys are determined by calculating the product of the weighted average of the *Bo* and *Md* values of the individual elements present in the alloy and their atomic fractions.

From such diagrams it is possible to determine the phases present in the alloy, the corresponding deformation mechanisms and values of Young’s modulus based on the Bo-*Md* ratio. The low values of the *E* correspond to the mechanism at the slip–twin boundary, where it is important to emphasize that when the beta phase has a higher stability, the slip mechanism prevails. Previous research has shown that higher values of *Bo* correspond to lower values of the Young’s modulus, and that at the same time these alloys are more resistant to corrosion [33].

**Figure 2:**
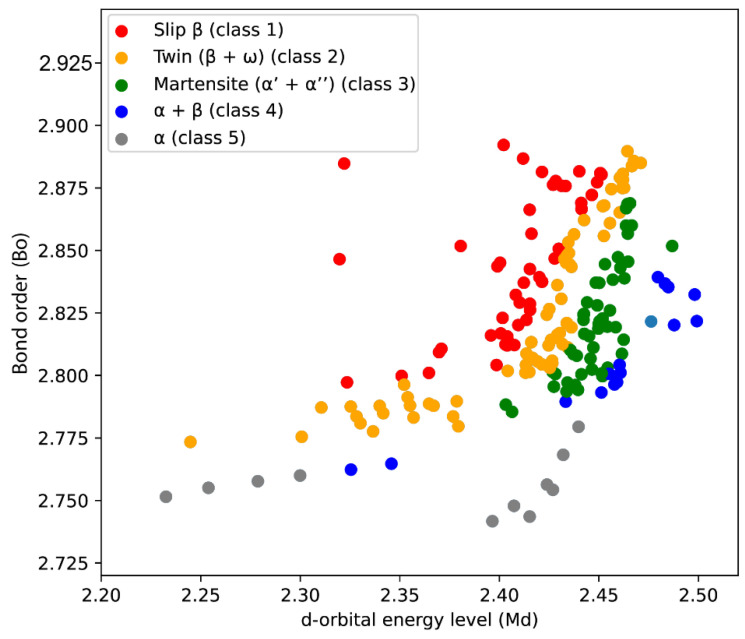
Classification of data based on d-orbital energy level (Md) and Bond order (Bo).

Figure 2 shows a scatterplot containing data of the modeled alloys and shows the stability phases, deformation mechanisms and Young’s modulus of different titanium alloys, depending on the strength of the covalent bond and the energy level of the d-orbit.

Using data from the literature [33], a colored dot plot was drawn where each point was assigned a specific color depending on where it is located in the extended *Bo-Md* diagram. The boundary for beginning of the martensitic transformation is marked as *M_s_* line (where *M_s_* is at room temperature, *M_s_* = RT), while the *M_f_*_1_ line represents the end of the martensitic transformation (where *M_f_*_1_ = RT) [33]. The boundary for the martensite is given as *M_f2_* curve [33]. The first group of points is marked in red (class 1), and these are the points that have a value greater than/equal to the corresponding values on the *M_s_* curve, and correspond to alloys that have a *β* phase that deforms by sliding. Among all phases, these are characterized by the lowest values of the Young’s modulus and have high strength. For our research, these stages are of great interest. The second group is characterized by the orange color (class 2) and these are the points that have values greater than/equal to the corresponding values on the *M_f_*_1_ curve, but less than the values on the *M_s_* curve and there is a *β + ω* phase. It is formed by cooling from high temperatures, and the ω phase is not particularly desirable because it can significantly affect the mechanical properties. The martensite *α*′ + *α*″ phase is marked with green color and represents values that are greater than or equal to the corresponding value on the *M_f_*_2_ curve, and less than the value on the *M_f_*_1_ curve. The next group, class 4, marked in blue, defines the range of the *α* + *β* phase and corresponds to values greater than or equal to the values on the *α* curve, which are at the same time smaller than the values on the *M_f_*_2_ curve. The last, fifth group, marked in gray, corresponds to the *α* phase and occupies a range of values smaller than those given on the *α* curve.

However, it is important to note that the results derived from the *Bo-Md* diagram may not be completely accurate and may present occasional deviations. This is primarily because different literature sources provide different data for this diagram, leading to potential variations in the predicted phase composition or transition temperatures. Moreover, factors such as thermomechanical treatments can alter the actual phase composition, further challenging the accuracy of the diagram. Therefore, the accuracy of phase composition predictions should be interpreted in light of these circumstances. Despite these limitations, the classification derived from *Bo-Md* plot proved to be useful in improving the performance of our model.

#### 3.2.2. Classification Based on the e/a Ratio

In the field of titanium alloys, it has been proven that the *E* of elasticity is influenced by the phase composition. Titanium alloys are categorized into three groups based on present phase as: *α*, *α* + *β,* and *β* alloys, where studies have shown that the *β* metastable phase is associated with the lowest values of the Young’s modulus. The *e/a* ratio (the ratio of free valence electrons to the number of atoms in the alloy) can be used as a parameter to design alloy with desirable phase stability and transitions. The stability of the *α* or *β* phase depends on the number of electrons per atom of the alloying element, and the appearance of the beta phase in the *e/a* ratio is associated with a value greater than 4.2.

**Figure 3:**
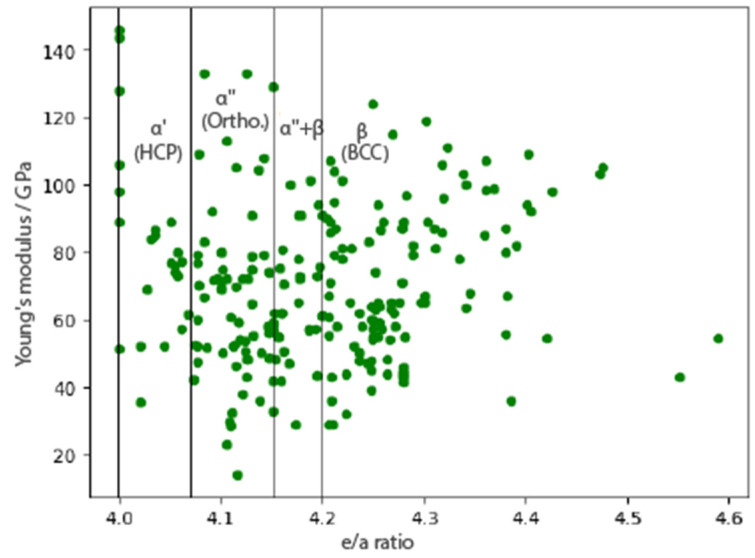
The value of the experimental Young’s modulus depending on the *e/a* ratio, and categorization of the present phase in Ti alloys regarding the *e/a* ratio.

In Figure 3, a scatter diagram was plotted using database values. The widespread dispersion of these values meant they did not offer highly reliable results for drawing definitive conclusions. This paper subsequently examines the relationship between the difference in atomic radius and *e/a*. Based on the theoretical model, it is possible to determine the deformation mechanism of the observed alloys, thereby predicting the present phases.

**Figure 4:**
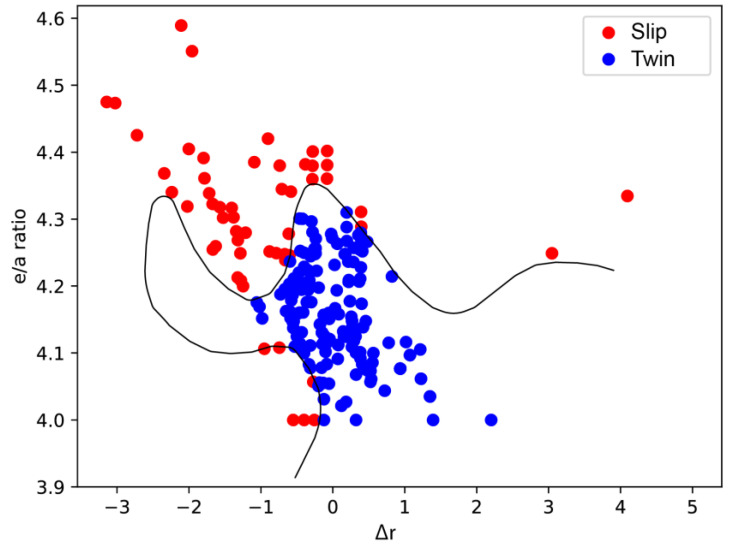
Dataset classification using the *e/a*–Δ*r* plot: differentiating between slip and twin mechanisms based on literature (borderline taken from the revised *e/a* versus Δ*r* diagram) [33].

Figure 4 illustrates a scatter diagram, leveraging binary classification derived from the literature [33]. The diagram showcases the correlation between the electron-atom ratio and differences in atomic radii. The diagram taken from the literature is the result of recent research and is designed to unify alloys produced by different methods, without taking sharp limits of the value of the *e/a* ratio to define the deformation mechanism presence. The data were classified in relation to the curve and marked in two categories. The first category of data is marked with blue dots and includes titanium alloys characterized by a twin mechanism, and the second group is marked with red dots and refers to alloys that are deformed by a slip mechanism. The stable *β* phase corresponds to the slip mechanism, while the metastable *β* phase is characterized by a mechanism at the slip + twin boundary. The line crosses some points in this diagram: if a data point’s center is outside the border line, it is denoted in red, indicating a slip mechanism. If the point’s center falls within the border line, it is colored blue, indicating a twin mechanism.

#### 3.2.3. Data Preprocessing

**Figure 5:**
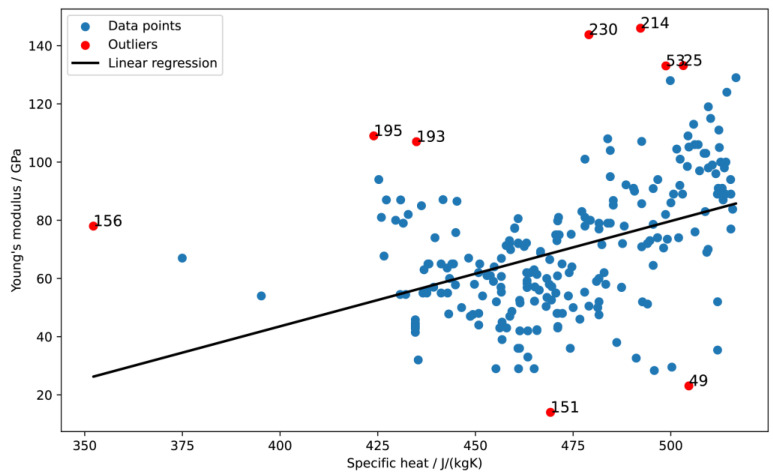
Detecting outliers in the dataset from the dependence of the experimental Young’s modulus and specific heat.

Using a variety of techniques to identify statistical outliers, it was found that linear regression was the most effective. Due to the apparent linearity between these parameters, this technique was employed specifically to analyze the relationship between the *E* and specific heat. As depicted in Figure 5, this method enabled the successful identification and elimination of outliers, thereby increasing the precision of the subsequent analysis. The cause of the presence of these outliers can be different, and most often due to different thermal and mechanical treatments on titanium alloys.

### 3.3. Model Performance

#### 3.3.1. Feature Selection

In the proposed model development process, we iteratively utilized performance metrics and a feature finding approach to identify key influential characteristics. These encompassed the weight percentages of several alloying elements such as titanium (Ti), niobium (Nb), zirconium (Zr), tantalum (Ta), Tin (Sn), iron (Fe), manganese (Mn), silicon (Si), molybdenum (Mo), and oxygen (O). Other significant features included the deviation of Young’s modulus from experimental data (Δ*E*), material density, the *e/a* ratio, two variables related to Mo equivalent ([*Mo*]*__eqB_*, [*Mo*]*__eqW1_*)*,* Bond order (*Bo*), d-orbital energy level (*Md*), specific heat, difference in atomic radii (Δ*r*), as well as classifications based on the *Bo-Md* diagram (group) and the *e/a* ratio (slip/twin group). The features were chosen due to their strong correlation with the target variable—the experimental *E*—while maintaining a low correlation among themselves. This selection helps prevent collinearity and thereby enhances the accuracy of the predictive model.

#### 3.3.2. Extra Trees Regressor

The Extra Trees Regressor model showed significant reliability in predicting the Young’s modulus of titanium alloys. Table 3 shows the performance applied to the train and test set, respectively. Key performance indicators such as mean absolute error (MAE) highlight the effectiveness of the model. It should be noted that the MAE of the proposed model fits well within the absolute error range reported in the existing literature. However, it is important to consider the inherent variability in the Young’s modulus due to thermomechanical treatments during alloy production. This variability is a limitation of the current model. In future work, inclusion of comprehensive thermomechanical treatment data for all alloys will be done which would significantly improve accuracy and performance of the used model.

**Figure 6:**
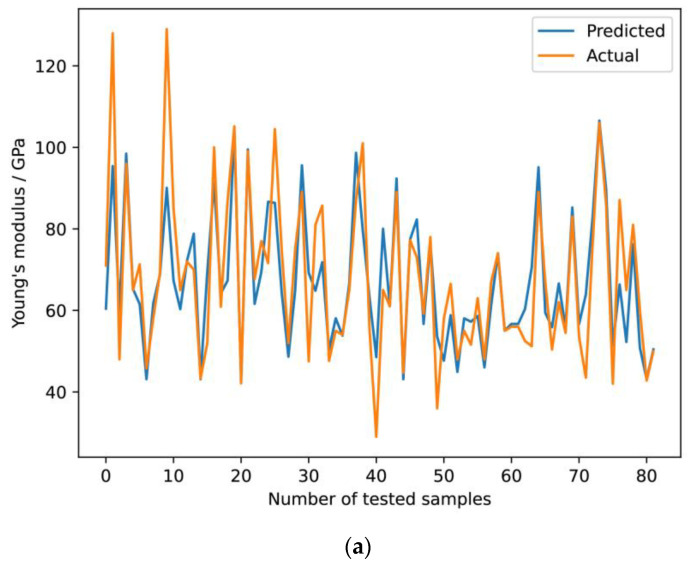

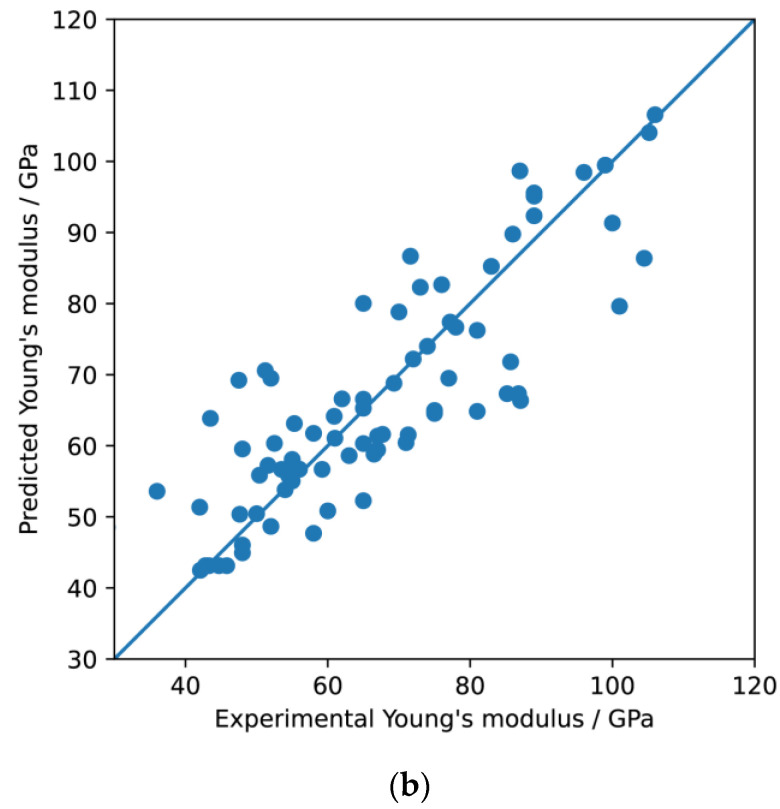
Predicted and actual value for the Young’s modulus (**a**) based on the number of tested samples (**b**) and its deviation from the linear dependence.

Figure 6a provides a graphical illustration of predicted versus actual results for Young’s modulus for test samples, while Figure 6b shows the deviation from linear dependence between predicted and actual values for *E*. A high degree of overlap indicates the accuracy of the used model. It is noticeable that the largest deviations between the predicted and actual values occur at the extremes of the *E*. This trend highlights the robustness of the model within conventional elasticity ranges and identifies areas of potential improvement for extreme elasticity values.

### 3.4. Variables Influencing the Young’s Modulus

**Figure 7:**
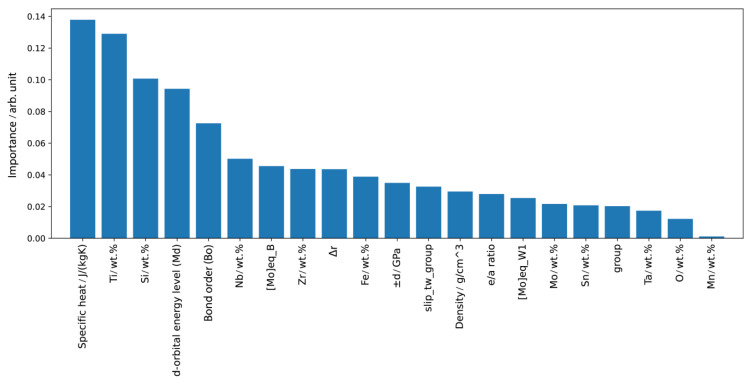
Feature importance for the experimental Young’s modulus.

The feature importance tool denotes the contribution of each feature or variable to the model’s predictive capability. As illustrated in Figure 7, these factors provide insight into how individual variables influence the model’s overall performance and help identify the key drivers of the modulus of elasticity predictions.

Specific heat is singled out as the most influential. This is particularly interesting and it is necessary to put a greater focus on the further interpretation because the previous researches do not have particularly many direct links with the modulus of elasticity, except for one, where V. Pekarek found relation between thermal properties and elastic modulus [38]:

$$c_{p}\cdot s=\frac{Ea}{1-4\cdot \sigma^{2}\;}\cdot \alpha$$

where *c_p_* is specific heat at constant pressure per kilogram (J/(kg·K)); *s* is density (kg/m^3^); *Ea* is Young’s modulus (GPa); *α* is linear coefficient of thermal expansion (1/K), and *σ* is Poisson’s ratio. The Einstein and Debye models, both quantum-mechanical in nature, define the change in molar heat capacity at varying temperatures, thereby explaining the indirect relationship between specific heat and the *E*. These models take into account the behavior of atomic vibrations, although from different perspectives. According to Einstein’s model, atoms vibrate independently of one another in a well-ordered state, similar to harmonic oscillators. This model effectively assumes that each atom within a solid oscillates independently. It is a good starting point, but it ignores atom-atom interactions. Debye’s model, on the other hand, accounts for atoms’ cooperative, or dependent, behavior. It proposes that atomic vibrations are not independent, but rather collective modes involving many atoms moving together. This model applies the theory to lower frequencies, resulting in a more accurate prediction of specific heat at low temperatures. These assumptions serve as the foundation for expressing the molar heat capacity, which is dependent on both the Einstein and Debye temperatures, as well as the temperature at which the molar heat capacity is measured. Notably, both Einstein and Debye temperatures are directly proportional to atom oscillation frequency. This oscillation frequency, which is fundamental to atomic behavior, can be calculated using *E*, thereby indirectly linking specific heat and *E* [39]. These models and their implications highlight the potential complexity of the interaction between specific heat and E, implying that the behavior of biocompatible Ti alloys can be influenced in a synergistic manner by a variety of factors. This complexity opens up exciting opportunities for future research into these interactions and their broader implications in material science and alloy design.

The next important variable is the mass content of titanium. The Young’s modulus of pure titanium is 110 GPa and as the main element in biocompatible titanium alloys, a higher mass fraction of this element often means higher values of the *E* [40]. Of course, in order to accurately define the impact, it is necessary to observe the exact composition of the alloy, as well as the thermomechanical processing methods, but in general it has been shown that a higher *E* corresponds to a higher mass fraction of the titanium alloy.

**Figure 8:**
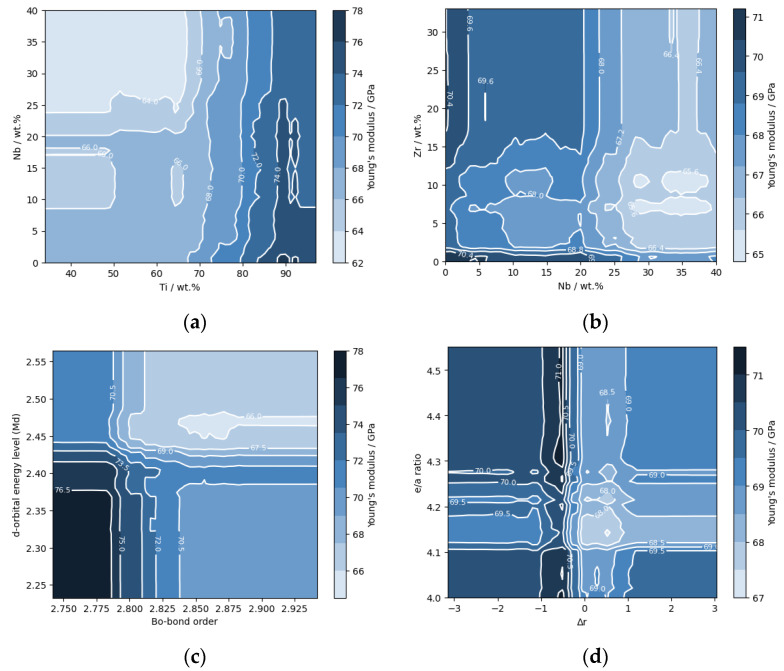
The influence of independent variables on the predicted Young’s modulus (**a**) niobium, (**b**) zirconium and niobium, (**c**) Bond order (*Bo*) and d-orbital energy level (*Md*), and (**d**) the *e/a* ratio and Δ*r*.

In our model, the mass fractions of various alloying elements, ordered by their feature importance, are as follows: Si, Nb, Zr, Fe, Mo, Sn, Ta, Mn, and O, along with Titanium. All of these, except for oxygen, serve as *β* stabilizers [15,37,41,42,43,44,45,46,47,48]. These elements, when incorporated in specific proportions, promote the formation of the *β* phase in titanium alloys, a phase noted for its lower *E*. Recently, extensive studies have been conducted to ascertain the contribution of each of these elements towards lowering the *E* modulus. For example, by examining the influence of Nb, it was found that in the Ti-33Nb-7Zr alloy, 33 wt.% of Nb leads to an extremely low *E* of 29 GPa [42]. Figure 8a,b show the influence of alloying elements on *E*.

The d orbital energy level (*Md*) and Bond order (*Bo*), as well as the ‘group’ feature (classification based on the diagram from Figure 2) were also identified as influential features (Figure 7). The d orbital energy level defines the bond strength and depends on the number of electrons in the d orbital, while the Bond order indicates the number of bonds between atoms. Both parameters are quantitative indicators of bond strength, with stronger bonds generally meaning higher *E* values. Diagrams constructed depending on these two parameters help to identify the current phases. Figure 8c) shows the predicted values for the *E* depending on these two factors, where it is noted that the minimum values are in the upper right corner.

Mo equivalents, particularly [*Mo*]*_eq_B_* and [*Mo*]*_eq_W1_*, were exhibited a positive effect on model performance, while other equivalents had a detrimental effect. By observing the formulas through which these two equivalents are expressed, it can be seen that in the first the coefficient with which Fe is multiplied is very high, while in the second it applies to Si, which can be connected to the strong influence of certain elements [46]:

[*Mo*]_*eq*_*B*_ = *Mo* + 0.67 *V* + 0.44 *W* + 0.28 *Nb* + 0.22 *Ta* + 2.9 *Fe* + 1.6 *Cr* + 0.77 *Cu* + 1.11 *Ni* + 1.43 *Co* + 1.54 *Mn* + 0.0 *Sn* + 0.0 *Zr* − 1.0 *Al* (wt%).

[*Mo*]_*eq*_*W*_ = *Mo* + 1.25 *V* + 0.59 *W* + 0.28 *Nb* + 0.22 *Ta* + 1.93 *Fe* + 1.84 *Cr* + 1.51 *Cu* + 2.46 *Ni* + 2.67 *Co* + 2.26 *Mn* + 0.3 *Sn* + 0.47 *Zr* + 3.01 *Si* − 1.47 *Al* (wt%).

The characteristics, the ‘*e/a* ratio’, ‘Δ*r*’ and ‘density’ were identified to possess a synergistic effect on model performance. In particular, the ‘*e/a* ratio’ serves as an indicator for the presence of *β* phase in alloys. When the ‘Δ*r*’, which indicates differences in atomic diameters, is related to the ‘*e/a* ratio’, it helps to determine the ‘slip or twin class’, thus improving the predictive ability of the model. It has been observed that higher density alloys typically correspond to higher values of *E*. The Δ*r* characteristic suggests that when the diameter differences are smaller, stronger bonds are likely to form, leading to a higher *E*. Figure 8d shows the fields of the lowest values of the *E* depending on these two factors, and the predictions coincide with the results shown in Figure 4.

It has been shown that the experimental *E* of elasticity variation (Figure 7), parameter: ±*d* (GPa) also contributes significantly to the performance of the model. Such variations can be attributed to a number of factors, the most influential of which are the sample preparation method and the thermomechanical treatments provided to the sample.

### 3.5. Predicting a Low Young’s Modulus for New Ti Alloys

#### 3.5.1. Four-Component Ti Alloys

Design of Experiments (*DoE*) represents a structured approach employed to plan and conduct experiments, aimed at understanding the influence of various factors—in this case, the alloy composition—on the target variable, which in this context is the experimental *E* (applying the created prediction model). Leveraging this approach, a matrix design was generated comprising 16,016 unique compositions, facilitating the investigation of the complex impact of three alloying elements on the *E*.

**Figure 9:**
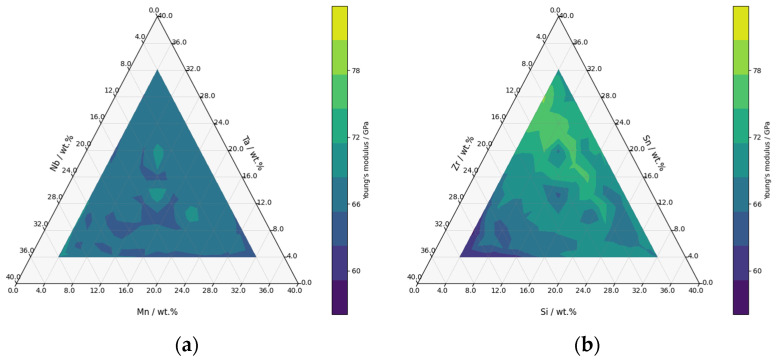
Ternary diagram showing predicted the Young’s modulus of the titanium alloy (**a**) Nb, Ta, and Mn; (**b**) Zr, Sn, and Si.

The resulting product is a set of ternary diagrams that display the mass fractions of various alloying elements, with a limitation that the maximum content of these elements in sum, does not exceed 40 wt.% (refer to Figure 9a,b). These diagrams are developed using a step size of 4 in the design matrix. Although this step size does not provide complete coverage of the diagram (from 0–0.1), it does facilitate a better visual representation with a field of predicted *E*.

Figure 9 presents two ternary diagrams, the first (a) depicting compositions with Nb, Ta, and Mn as alloying elements, and the second (b) presenting those with Zr, Sn, and Si as alloying elements. The dark purple regions on these diagrams are indicative of areas where Young’s modulus falls below 60 GPa. This graphical representation offers a clear demarcation of areas associated with lower *E*, providing useful guidelines for the development of novel titanium alloys.

#### 3.5.2. Monte Carlo Simulations for the Design of Multicomponent Alloys

The Monte Carlo simulation method was used to generate ten million alloy samples under controlled conditions to achieve multicomponent alloys with desired *E* values. This method yielded several promising alloys, four of which are presented in Table 4. We set the oxygen content to a maximum of 0.78 wt.% in line with the upper limit found in the literature.

The main elements of these alloys are titanium, zirconium, tin, manganese, tantalum, and niobium, which could relate to the studies of the most influential variables of the biocompatible Ti alloys in past research. Without concrete experimental data, it is currently difficult to specify the phases or intermetallic compounds that may form in these proposed compositions. While the specific roles and interactions of these elements within the alloy matrix are subjects for future experimental studies, their proportions in the given samples are computed based on the objectives of achieving desired Young’s modulus values as indicated in Table 4. The predicted Young’s modulus values serve as preliminary indicators of the anticipated mechanical properties of these alloys, offering a direction for future experimental validations and investigations.

## 4. Conclusions

The objective of the present study was to analyze trends in the elastic behavior of biocompatible and low *E* Ti alloys using machine learning. The proposed computational approach for predicting the composition and *E* of biocompatible Ti alloys was composed of the following steps: (1) establishing a database, (2) conducting an exploratory data analysis, (3) building new feature classifications according to solid scientific principles, and (4) identifying an appropriate regression model. The Extra Tree Regression model is implemented as the most efficient in predicting the *E*. The obtained results determined the specific heat as the most influential parameter on the *E* of low-modulus biocompatible Ti alloys. Even though this finding is supported with limited data in the literature, it contributes to the expansion of current knowledge in the field of biocompatible Ti alloys.

The construction of the *Bo-Md* diagram predicts the dependence between the phase stability and Young’s modulus of Ti alloys, as well as the deformation mechanisms corresponding to the given phases. Particularly, numerous alloys are placed on the *α* and *α* + *β* border when the *Bo* value is greater than 2.77. These metastable *β* alloys are deformed by a slip–twin mechanism, which induces better mechanical properties as well as increasing plasticity and fatigue resistance.

Furthermore, an *e/a*–Δ*r* plot was used to define the deformation mechanism, using two semi-empirical parameters. The first method proved to be more valid, especially for more complex compositions. Monte Carlo simulation was leveraged to predict the composition of multicomponent Ti alloys. The proposed computational approach could accelerate improvement of existing and development of novel low-modulus biocompatible Ti alloys.

## Figures and Tables

**Table 1:** Classification of alloying elements in titanium alloys.

*α*-Stabilizers	Neutral Stabilizers	*β*-Stabilizers
Al, O, N, C, Ga	Sn, Zr, Hf,	Mo, V, Nb, Ta, Fe, Cr, Mn, Ni, Co, W

**Table 2:** Classification of alloying elements in titanium alloys depending on cytotoxicity.

Non-Cytotoxic Elements	Neutral Elements	Cytotoxic Elements
Ti, Nb, Zr, Ta, Ru, Sn	W, Fe, Mn, Si, O, N, C, Hf	Pt, V, Al, Ni, Co, Cu, Cr

**Table 3:** Performance applied to the training and test set.

	MAE	R^2^	MSE	MAX	MAPE
Training set	0.633	0.989	5.662	14.5	0.011
Test set	7.722	0.699	119.6	38.9	0.118

**Table 4:** Values of various parameters for modeled multicomponent alloys.

Parameter	Unit	Sample ID			
		2165217	2351451	3295558	9044251
Ti	wt.%	17.90	18.59	59.57	50.76
O	wt.%	0.56	0.17	0.092	0.099
Nb	wt.%	18.27	24.38	18.92	14.42
Zr	wt.%	12.03	13.15	3.03	2.32
Ta	wt.%	19.27	13.65	1.30	7.10
Sn	wt.%	18.37	28.94	2.71	5.04
Mn	wt.%	8.74	0.41	7.55	8.34
Si	wt.%	0.53	0.22	1.05	0.045
Mo	wt.%	2.68	0.27	5.21	11.24
Fe	wt.%	1.65	0.22	0.57	0.62
[*Mo*]*_eq_B_*	/	30.29	11.35	24.09	31.49
[*Mo*]*_eq_W_*_1_	/	47.74	26.97	34.36	39.63
Bo-bond order	/	2.04	2.32	2.23	1.74
d orbital energy level *(Md)*	/	1.64	1.82	1.94	1.50
Specific heat	(J/kgK)	303.39	309.07	388.60	309.47
Δ*r*	/	−4.82	−1.95	−1.57	−2.02
Predicted Young’s modulus	GPa	67.29	67.14	64.63	64.57

## Data Availability

The data presented in this study are available on request.

## Appendix A

**Table A1:** Metrics of analyzed variables.

Parameter	Unit	Count	Mean	Std	Min	50%	Max
Ti	wt.%	243.0	69.43	13.90	20.00	68.00	96.94
Ti	at.%	243.0	81.10	9.39	32.47	81.22	98.45
Nb	wt.%	243.0	17.05	13.52	0.00	15.50	45.19
Nb	at.%	243.0	10.96	9.50	0.00	9.91	33.45
Zr	wt.%	243.0	4.53	5.82	0.00	3.00	40.00
Zr	at.%	243.0	2.94	4.10	0.00	2.03	34.07
Ta	wt.%	243.0	2.83	7.67	0.00	0.00	70.00
Ta	at.%	243.0	1.10	3.41	0.00	0.00	38.17
Sn	wt.%	243.0	2.59	4.20	0.00	0.00	20.00
Sn	at.%	243.0	1.26	2.03	0.00	0.00	9.16
Fe	wt.%	243.0	0.72	1.93	0.00	0.00	10.00
Fe	at.%	243.0	0.68	1.80	0.00	0.00	9.40
Mn	wt.%	243.0	0.59	2.59	0.00	0.00	17.65
Mn	at.%	243.0	0.52	2.29	0.00	0.00	15.71
Si	wt.%	243.0	0.03	0.16	0.00	0.00	1.25
Si	at.%	243.0	0.06	0.31	0.00	0.00	2.37
Mo	wt.%	243.0	2.20	4.47	0.00	0.00	38.08
Mo	at.%	243.0	1.27	2.65	0.00	0.00	25.31
O	wt.%	243.0	0.06	0.15	0.00	0.00	0.78
O	at.%	243.0	0.20	0.55	0000	0.00	2.81
N	wt.%	243.0	0.00	0.01	0.00	0.00	0.05
N	at.%	243.0	0.00	0.02	0.00	0.00	0.19
C	wt.%	243.0	0.00	0.00	0.00	0.00	0.02
C	at.%	243.0	0.00	0.01	0.00	0.00	0.06
Young’s modulus, exp	GPa	243.0	69.63	23.31	14.00	66.50	146.0
±d	GPa	243.0	0.70	2.56	0.00	0.00	26.70
Elongation	%	94.0	14.49	10.92	0.09	13.75	42.00
Max tensile strength	MPa	100.0	884.94	345.74	246.00	851.50	2093.0
Yield strength	MPa	97.0	777.29	266.27	156.00	787.00	1785.0
Hardness	HV	69.0	305.71	89.40	145.00	312.00	490.0
HT1: T	C	102.0	1025.54	211.29	450.00	1000.00	1400.0
HT2: T	C	46.0	761.67	239.25	30.00	879.85	1000.0
Weighted molar mass sum	/	243.0	64.24	11.08	48.24	64.03	141.0
Density	g/cm^3^	243.0	5.87	0.97	4.60	5.67	13.0
*e/a* ratio	/	243.0	4.19	0.11	4.00	4.19	4.59
Bo-bond order	/	243.0	2.83	0.04	2.74	2.82	3.00
[Mo]eq_B	/	243.0	10.56	6.41	0.00	10.24	41.47
[Mo]eq_Z	/	243.0	12.19	6.16	0.00	12.45	42.80
[Mo]eq_W1	/	243.0	13.27	5.68	1.50	12.78	41.47
[Mo]eq_W2	/	243.0	11.22	4.30	1.57	11.25	40.86
[Mo]eq_chen	/	243.0	10.50	6.36	0.00	10.17	41.44
d-orbital energy level (Md)	/	243.0	2.43	0.05	2.23	2.43	2.61
Specific heat	J/(kgK)	243.0	472.09	27.29	352.23	470.91	516.7
Bulk modulus	GPa	243.0	121.26	7.03	105.24	120.52	160.7
Shear modulus	GPa	243.0	43.00	2.00	37.43	42.513305	52.8
ΔL1	/	243.0	4.09	4.47	−0.16	3.22	31.7
Δ*r*	/	243.0	−0.18	0.83	−3.14	−0.15	4.10
Y_ls_coef	GPa	243.0	60.55	26.33	5.56	58.28	177.2
Y_th	/	243.0	116.75	7.60	95.62	114.82	169.0
